# A Rare Mono-Rhamnolipid Congener Efficiently Produced by Recombinant *Pseudomonas aeruginosa* YM4 via the Expression of Global Transcriptional Regulator *irrE*

**DOI:** 10.3390/molecules29091992

**Published:** 2024-04-26

**Authors:** Xinying Wang, Dongmei Li, Shenghui Yue, Zhangzhong Yuan, Shuang Li

**Affiliations:** 1 College of Biotechnology and Pharmaceutical Engineering, Nanjing Tech University, Nanjing 211816, China; 202161118022@njtech.edu.cn (X.W.); 202362182196@njtech.edu.cn (D.L.); 2 Research Institute of Petroleum Engineering and Technology, Sinopec Shengli Oilfield, Co., Ltd., Dongying 257000, China; yueshenghui.slyt@sinopec.com (S.Y.); yuanzhangzhong.slyt@sinopec.com (Z.Y.)

**Keywords:** rhamnolipid, *Pseudomonas aeruginosa*, biosurfactant, *irrE*, global regulator

## Abstract

Rhamnolipids (RLs) are widely used biosurfactants produced mainly by *Pseudomonas aeruginosa* and *Burkholderia* spp. in the form of mixtures of diverse congeners. The global transcriptional regulator gene *irrE* from radiation-tolerant extremophiles has been widely used as a stress-resistant element to construct robust producer strains and improve their production performance. A P_rhlA_-*irrE* cassette was constructed to express *irrE* genes in the *Pseudomonas aeruginosa* YM4 of the rhamnolipids producer strain. We found that the expression of *irrE* of *Deinococcus radiodurans* in the YM4 strain not only enhanced rhamnolipid production and the strain’s tolerance to environmental stresses, but also changed the composition of the rhamnolipid products. The synthesized rhamnolipids reached a maximum titer of 26 g/L, about 17.9% higher than the original, at 48 h. The rhamnolipid production of the recombinant strain was determined to be mono-rhamnolipids congener Rha–C_10_–C_12_, accounting for 94.1% of total products. The critical micelle concentration (CMC) value of the Rha–C_10_–C_12_ products was 62.5 mg/L and the air-water surface tension decreased to 25.5 mN/m. The Rha–C_10_–C_12_ products showed better emulsifying activity on diesel oil than the original products. This is the first report on the efficient production of the rare mono-rhamnolipids congener Rha–C_10_–C_12_ and the first report that the global regulator *irrE* can change the components of rhamnolipid products in *Pseudomonas aeruginosa*.

## 1. Introduction

Rhamnolipids are a glycolipid biosurfactant and can be applied in daily chemistry [1], food, agriculture [2], medicine [3], and microbial enhanced oil recovery (MEOR) [4]. At present, *Pseudomonas aeruginosa* is among the most potent and best characterized native rhamnolipids producers. The natural rhamnolipid products show rich structural diversity. More than 60 rhamnolipid congeners have been discovered. Among them, the difference in the number and length of the hydrophilic rhamnolipid groups and hydrophobic fatty acid chains makes the structure of rhamnolipids diverse [5]. In terms of rhamnolipids produced by *Pseudomonas aeruginosa* strains, they are mainly divided into four types: Rha–C_10_, Rha–C_10_–C_10_, Rha–Rha–C_10_, and Rha–Rha–C_10_–C_10_. The variation of congeners and contents of rhamnolipid products have significant influence on their physicochemical properties and subsequent application [6,7].

Rhamnolipids have remarkable tensioactive and emulsifying properties. Compared to chemical surfactants, rhamnolipids have the advantages of being non-toxic, environmentally friendly, and biodegradable. However, the use of rhamnolipids is limited. An important factor hindering the large-scale application of rhamnolipids at present is their high production cost. It is necessary to reduce the cost, increase rhamnolipid production and expand their applications. To enhance the efficiency (e.g., titer, rate, and yield) of rhamnolipid production, a lot of work has been carried out, including strain improvement and fermentation optimization [8,9,10,11]. The amphiphilic structure of rhamnolipids is comparable to that of cell membranes. Rhamnolipids intercalate into cell membranes via interaction with lipopolysaccharide-binding proteins (LBPs), causing toxicity to cells [12,13,14]. Considering the stress and toxicity caused to the producer cells by the high concentration of rhamnolipid accumulation in fermentation broth, it is worth trying to improve the stress tolerance of producers to obtain a higher rhamnolipid titer.

The global transcriptional regulator *irrE* (or *PprI*) of *Deinococcus radiodurans*, a protein of 35-kD, plays a central regulatory role in multiple DNA damage repair and protection pathways in response to radiation stress [15,16]. As a global regulator of extreme stress responses, *irrE* has been used as a stress-resistant element to build more robust production strains. The heterologous expression of *irrE* and its mutants in *Escherichia coli* [17], *Saccharomyces cerevisiae* [18], *Zymomonas mobilis* [19], *Sphingomonas* sp. [20], and *Arthrobacter simplex* [21] can cause significant changes in the host transcriptome and proteome, and enhance tolerance to various stresses such as acid, salt, organic solvents, inhibitors in lignocellulose hydrolysates, and high temperatures.

**Figure 1:**
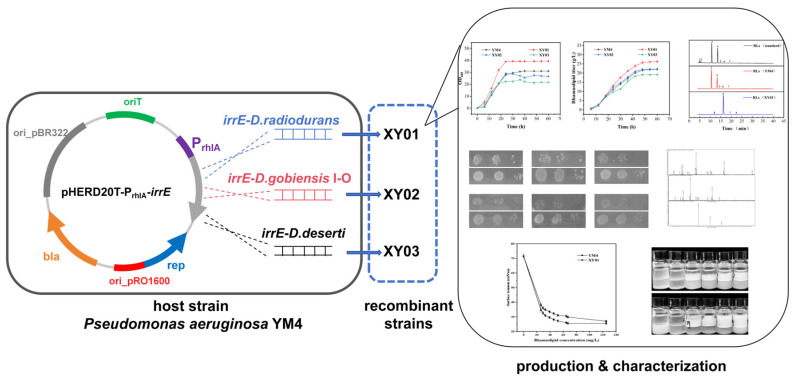
Experimental design process diagram.

Our original intention was to obtain a more robust rhamnolipid-producing strain by heterologous expression of the global transcriptional regulator *irrE*. Thus, the global transcriptional regulator *irrE* from *Deinococcus* species was applied in rhamnolipid producer *Pseudomonas aeruginosa* YM4. Unexpectedly, a rare mono-rhamnolipids congener could be synthesized in large quantities in one of the *irrE*-expressing strains. Furthermore, the new products were determined and characterized, as shown in Figure 1. This work emphasizes that the global regulator *irrE* of *D. radiodurans* played a complex regulatory role in *Pseudomonas aeruginosa* YM4, which provides new approaches for improving strains and developing new rhamnolipid products.

## 2. Results

### 2.1. Screening for Suitable Promoters

**Figure 2:**
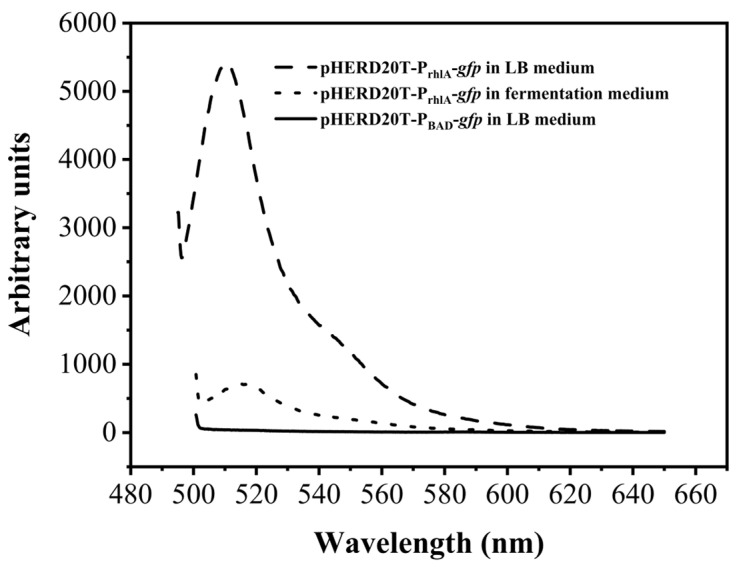
The fluorescence of recombinant YM4 cells containing the P_rhlA_-*gfp* cassette and P_BAD_-*gfp* cassette cultivated in LB medium and rhamnolipid fermentation medium without the addition of any inducer.

In the original plasmind pHERD20T, the target gene is under the control of the P_BAD_ promoter. In general, 10 g/L of L-arabinose is needed to induce the expression of the target gene [22]. Considering the cost of the inducer arabinose, it is more beneficial to replace the induced promoter with a constitutive promoter. The acyltransferase RhlA synthesizes 3-(3-hydroxyalkanoyloxy)alcanoic acids (HAAs) by esterification of two 3-hydroxyfatty acids, and this is often seen as the first step in rhamnolipid synthesis in *P. aeruginosa* [23]. In our work, the original promoter P_BAD_ was replaced with the promoter of *rhlA* (P_rhlA_), and P_rhlA_ was used to control the expression of the desired gene. Taking *gfp* as the reporter gene, the expression cassette P_rhlA_-*gfp* was constructed, and the transcription intensity of P_rhlA_ in recombinant strain *P. aeruginosa* YM4 was identifed by measuring the fluorescence of GFP using a fluorescence spectrophotometer. Recombinant strain *P. aeruginosa* YM4 containing the original expression cassette P_BAD_-*gfp* was used as the control. Figure 2 shows the whole-cell fluorescence of recombinant strain cells containing the P_rhlA_-*gfp* cassette and P_BAD_-*gfp*. It shows that the *gfp* gene under the control of P_rhlA_ in recombinant strain YM4 could be well expressed both in lysogeny broth (LB) medium and in the rhamnolipid fermentation medium without the addition of any inducer. No fluorescence was detected in the control group (P_BAD_-*gfp* cassette). It confirmed that P_rhlA_ was an efficient constitutive promoter.

### 2.2. Effects of the Global Transcriptional Regulator IrrE on Growth and Rhamnolipds Titer in Batch Fermentation

The global transcriptional regulator *irrE* from a different source was constitutively expressed in recombinant strain YM4 by constructing the P_rhlA_-*irrE* cassette in plasmid pHERD20T; then, the recombinant YM4 cells containing the P_rhlA_-*irrE* cassette were named as XY01 (*irrE* from *D. radiodurans*), XY02 (*irrE* from *D. gobiensis* I-O), and XY03 (*irrE* from *D. deserti*). Taking the starting strain YM4 as the control, all strains were cultivated in rhamnolipid fermentation medium. The cell growth was monitored by measuring the optical density at 600 nm (OD_600_) and the rhamnolipid titer was determined directly using HPLC–ELSD.

**Figure 3:**
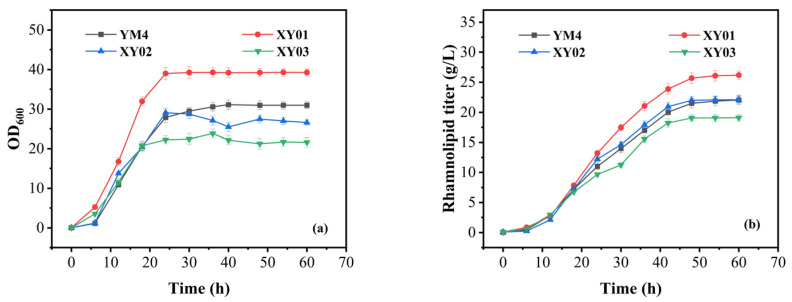
Fermentation performance of strain YM4 and recombinant YM4 cells containing the P_rhlA_-*irrE* cassette in batch fermentation. (**a**) The growth of strains; (**b**) the rhamnolipid production of strains. All strains were cultivated in rhamnolipid fermentation medium. The error bars represent standard deviation values of three independent experiments (*n* = 3).

Figure 3a shows the cell growth of all strains. The growth of XY01 was the best. Compared with strain YM4, the cell density (OD_600_) of XY01 significantly increased to 39.28, resulting in a 27.4% increase in biomass. However, the growth of XY03 decreased significantly, and the biomass decreased by approximately 23.5%. Figure 3b shows the rhamnolipid production of all strains. We can see that strain XY01 had the highest titer, and the rhamnolipid titer reached 26 g/L at 48 h, which was about 17.9% higher than that of strain YM4. The productivity of strain XY01 was about 541.7 mg/(L·h).

**Figure 4:**
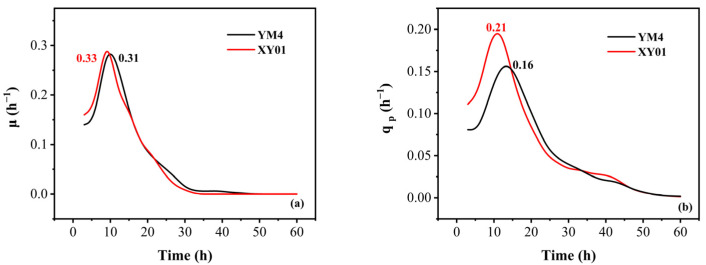
The variation of kinetic parameters (µ, q_p_) of strain XY01 and YM4. (**a**) The specific growth rate (µ, h^−1^); (**b**) the specific rhamnolipid production rate (q_p_, h^−1^).

The parameters μ and q_p_ of strain XY01 and strain YM4 are shown in Figure 4. The maximum specific growth rate (μ_max_) of XY01 was 0.33 h^−1^ at 10 h, which was 6.5% higher than that of YM4. It was noteworthy that the maximum specific product synthesis rate (q_pmax_) of XY01 was 0.21 h^−1^ at 13 h, which was 31.3% higher than that of YM4.

These results suggested that the heterologous expression of the global transcriptional regulator *irrE* from *D. radiodurans* could improve cell growth, which in turn promoted rhamnolipid production. Thus, strain XY01 was chosen to be further investigated.

### 2.3. IrrE of D. radiodurans Improved the Robustness of P. aeruginosa

**Figure 5:**
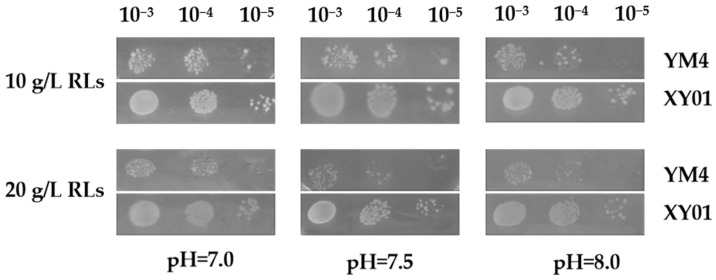
Response of cells to stress environment. The growth phenotypes of strain XY01 and YM4 were evaluated on LB agar plates supplemented with 10 g/L or 20 g/L rhamnolipids and with pH adjusted to 7.0, 7.5, and 8.0. All plates were photographed after 12 h incubation at 37 °C.

During the batch fermentation in flasks, the rhamnolipids accumulated to high concentration, and the pH of the broth gradually increased from 7.0 to 8.0. This became a stressful environment for producer cells. The tolerance of strain XY01 of rhamnolipids and an alkaline environment were tested by spot assay. As shown in Figure 5, with the increase of rhamnolipid concentration and pH, the growth of original strain YM4 was significantly inhibited. However, the recombinant strain XY01 containing heterologous *irrE* exhibited better growth performance under the same stress conditions; it grew faster or was numerically more dominant than the control strain YM4. These results indicated that heterologous expression of *irrE* gene in *P. aeruginosa* could enhance the robustness of *P. aeruginosa* and improve its tolerance of stressful environments.

### 2.4. Characterization of Rhamnolipids

#### 2.4.1. HPLC-ELSD Analysis of Rhamnolipids

**Figure 6:**
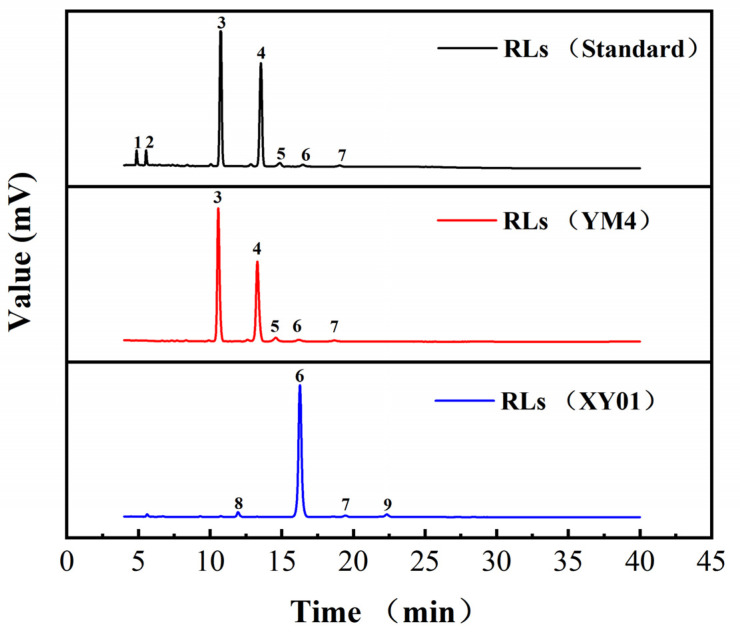
HPLC-ELSD profiles of rhamnolipids produced from strain YM4 and XY01. Rhamnolipids standard (R90, Lot number: A79125126058) was obtained from AGAE Technologies (Corvallis, OR, USA).

In our previous work, strain YM4 was an efficient di-rhamnolipid producer, and di-rhamnolipids accounted for 64.8% and 85.7% of total products with soybean oil and glycerol as carbon sources, respectively [24]. HPLC-ELSD profiles of the rhamnolipid products are shown in Figure 6, and the structural composition of rhamnolipid products is shown in Table 1. A total of nine rhamnolipid congeners were detected. The products of strains YM4, XY02, and XY03 exhibited similar components and contents, with di-rhamnolipids (Rha_2_–C_10_–C_10_) (10.56 min) and mono-rhamnolipids (Rha–C_10_–C_10_) (13.28 min) as major components, and together accounting for 97–98% of total products. The rhamnolipids of strain XY01 were significantly different. The congener of peak 6 (16.28 min) was the major component, accounting for 94.1%. However, the congener of peak 6 (16.28 min) also appeared in the rhamnolipids standard and products of YM4 as a rare component, accounting for 0.5–2%. The congeners of peak 8 (11.95 min) and peak 9 (22.33 min) were unique new components of rhamnolipids produced by strain XY01. These congeners were not found in the original products of strain YM4. As the proportion of such congeners was low to less than 3%, we performed no in-depth research on them. This indicated that global regulator *irrE* from *D. radiodurans* could regulate the biosynthesis of rhamnolipids and generate novel rhamnolipid products in strain *P. aeruginosa* YM4. However, *IrrE* from *D. gobiensis* I-O and *D. deserti* could not significantly influence the rhamnolipid composition in recombinant strains. The congener of peak 6 (16.28 min) was further determined by HPLC-MS.

#### 2.4.2. HPLC-MS Analysis of Rhamnolipids

**Figure 7:**
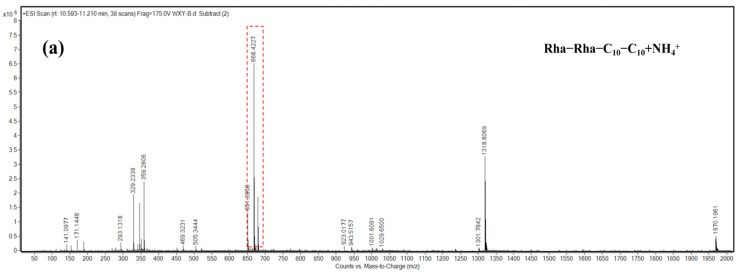

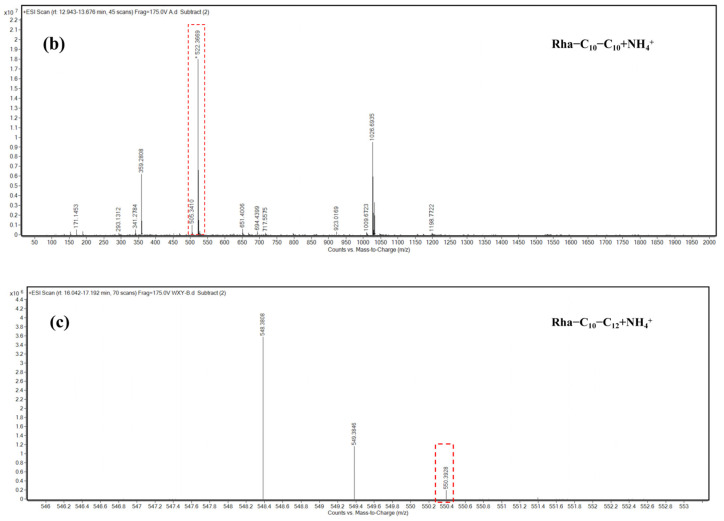
HPLC-MS characterization of rhamnolipid congeners of peaks 3, 4, and 6. (**a**) Congener of peak 3 mass spectra of the fragmented pseudomolecular ion at *m*/*z* 668 of Rha_2_–C_10_–C_10_ and the daughter ions generated by fragmentation; (**b**) congener of peak 4 mass spectra of the fragmented *m*/*z* 522 pseudomolecular ions of Rha–C_10_–C_10_ and the daughter ions generated by fragmentation; (**c**) congeners of peak 6 mass spectra of the fragmented pseudomolecular ion at *m*/*z* 550 of Rha–C_10_–C_12_ and the daughter ions generated by fragmentation.

The molecular weight information of the main components that appeared as peaks 3, 4, and 6 was obtained by mass spectrometry analysis. According to the structural characteristics of rhamnolipid congeners and the *m*/*z* of each component, the probable structure represented by each peak was deduced. As presented in Figure 7, the congeners of peak 3 and peak 4 were deduced to be Rha_2_–C_10_–C_10_ and Rha–C_10_–C_10_, with the major [M + NH_4_]^+^ peak at *m*/*z* 668 and 522, respectively. The congener of peak 6 was deduced to be Rha–C_10_–C_12_, with the [M + NH_4_]^+^ peak at *m*/*z* 550.

#### 2.4.3. Fourier-Transform Infrared (FTIR) Analysis of Rhamnolipids

**Figure 8:**
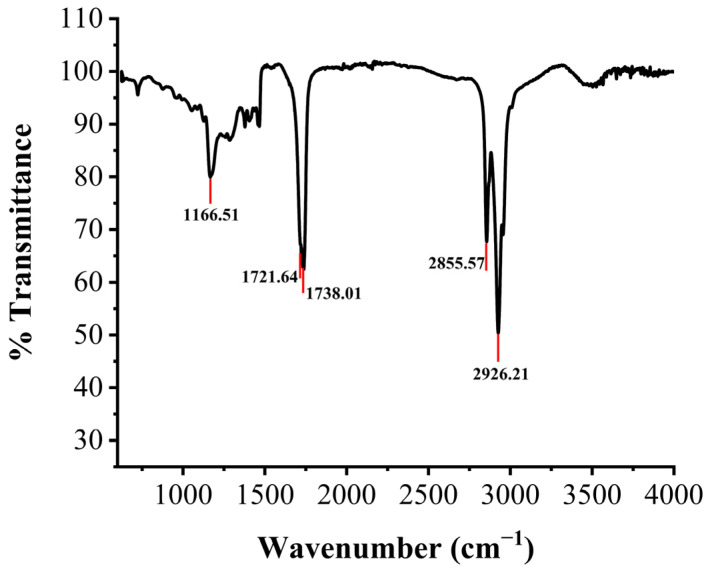
FTIR spectrum profile of rhamnolipids produced by strain XY01.

The types of functional groups and chemical bonds in the structure of an unknown compound are relatively easy to determine by the FTIR spectra. To further confirm the structure of the new components, the purified products of XY01 were analyzed by infrared spectroscopy. As shown in Figure 8, there were characteristic absorbance bands at 3500 cm^−1^ (–OH group stretching due to hydrogen bonding), 1738 cm^−1^ (C=O stretching of the ester linkage), 1721 cm^−1^ (C=O stretching carboxylate anion), 1319–1052 cm^−1^ (C–O–C and C–OH stretching in the rhamnose), 2926 cm^−1^, and 2855 cm^−1^ (the aliphatic bonds –CH_3_, –CH_2_, and –C–H stretching). The absence of bands at 3300–3000 cm^−1^ demonstrated the absence of H–C=C–H bonds. This indicates that the structure of rhamnolipid XY01 did not contain unsaturated fatty acids.

#### 2.4.4. The Critical Micelle Concentration (CMC) of Rhamnolipids

**Figure 9:**
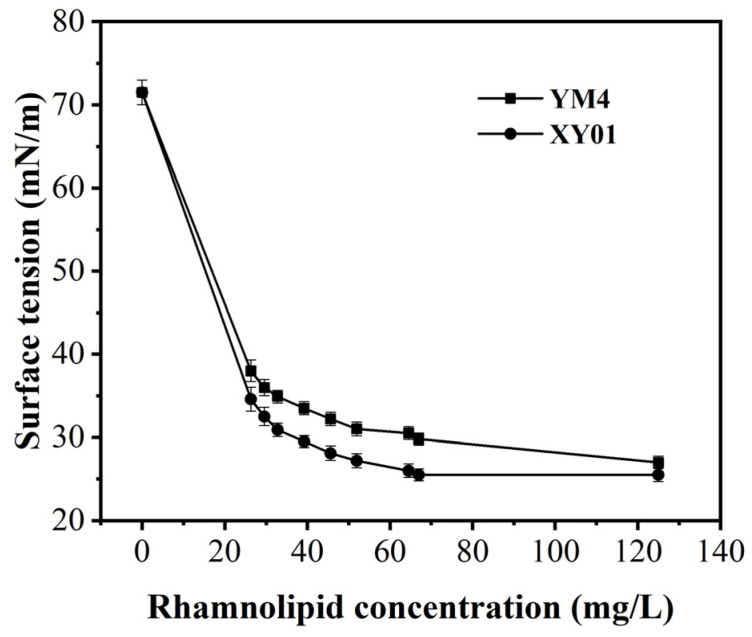
Surface tension changes and CMC values of rhamnolipids produced by strain XY01 and YM4. The error bars represent standard deviation values of three independent experiments (*n* = 3).

CMC is the lowest concentration of surfactant needed to form micelles. In our previous work, the CMC values of rhamnolipids were related to their components, ranged between 57 and 75 mg/L [25]. As demonstrated in Figure 9, both rhamnolipids were able to significantly reduce the surface tension of water; the mono-rhamnolipid products of strain XY01 exhibited a lower CMC value of 62.5 mg/L and a lower surface tension of 25.5 mN/m.

#### 2.4.5. Emulsifying Activity of Rhamnolipids on Diesel Fuel

**Figure 10:**
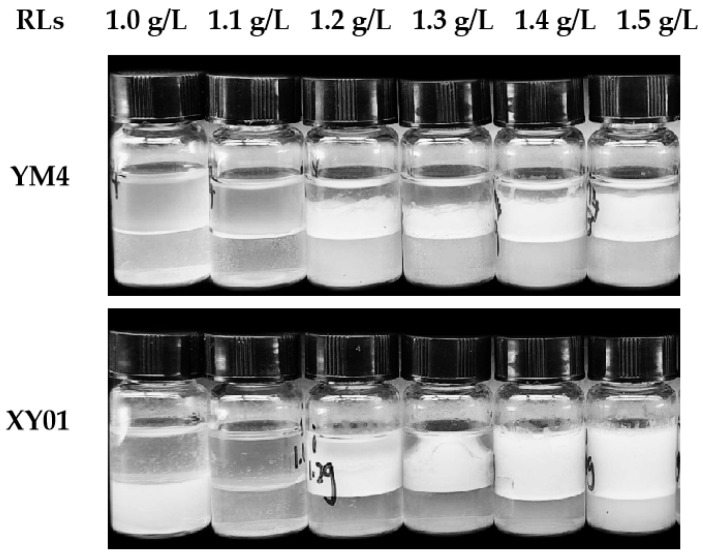
The emulsifying activity of rhamnolipids produced by XY01 and YM4 on diesel oil at different concentrations.

It has been shown that the concentration and structural composition of rhamnolipids are important factors in emulsifying activity. Since the rhamnolipid concentrations in the tests were much higher than the CMC values, the emulsifying activity was greatly affected by the structural composition of rhamnolipids rather than rhamnolipid concentrations. The effect of rhamnolipid products on the emulsion layer formed from diesel oil is shown in Figure 10. It shows that the mono-rhamnolipid products (Rha–C_10_–C_12_) of strain XY01 exhibited greater emulsifying activity on diesel oil. At the concentration of 1.5 g/L, the EI_24_ of XY01 rhamnolipids was 72.7%, much higher than that of YM4 rhamnolipids (40.9%).

## 3. Discussion

*IrrE* is a well-conserved global regulator in the *Deinococcus* species and governs gene transcription in the DNA damage response (DDR), providing extraordinary radiation resistance to the genus *Deinococcus*. Until now, *IrrE* from *Deinococcus radiodurans* and its mutations have been widely used as a tolerance-enhancer for *Zymomonas mobilis* [19], *Saccharomyces cerevisiae* [18], and *E. coli* [26] for ethanol production under various stresses. In this work, the functions of the global regulator *irrE* from three *Deinococcus* species were tested, labeled as *irrE*01 from *Deinococcus radiodurans, irrE*02 from *Deinococcus gobiensis I-O,* and *irrE*03 from *Deinococcus deserti.* The homologs irrE02 and irrE03 shared 65.2% and 65.1% sequence identity with irrE01, respectively. Studies have shown that *irrE*02 and *irrE*03 could restore radiation resistance in the *D. radiodurans irrE* mutant (∆*irrE*) and even *irrE*02 showed a stronger protection phenotype against UV radiation than *irrE*01 [27,28]. There is no application of *irrE*02 and *irrE*03 for enhancing stress tolerance. We tried using *irrE* from *Deinococcus gobiensis I-O* and *Deinococcus deserti* as stress-resistant elements for increasing the efficiency of microbial cell factories for the first time, but unfortunately we did not obtain positive results.

*IrrE* from *D. radiodurans* has been used as an heterologous global regulator in the model strain *Pseudomonas aeruginosa* PAO1 to improve electricity production in microbial fuel cells (MFCs). The results indicated that *irrE* significantly affected substrate utilization profiling, improving cell growth and cell tolerance to various stresses, and *irrE* led to many differently expressed genes, which were responsible for phenazines’ core biosynthesis, biofilm formation, QS systems, transcriptional regulation, glucose metabolism, and general stress response [29]. Additionally, heterologous expression of *irrE* of *D. radiodurans* had been shown to improve the acid tolerance of pollutant-degrading bacterium *Pseudomonas putida* S16 and enhance its viability and biodegrading activities in bioremediation of acidic wastes [30]. Similarly, in our work, *irrE* from *D. radiodurans* was found to be effective in increasing the rhamnolipid production of *Pseudomonas aeruginosa* YM4, as well as cell growth and cell tolerance to stress. Therefore, the global regulator *irrE* can be used as viable tools for improving stress resistance and production efficiency of genus *Pseudomonas*.

Rhamnolipids are composed of one or two rhamnose molecules linked to one or mostly two 3-hydroxyfatty acids of varying chain lengths. According to the the number of L-rhamnose residues, the rhamnolipids can be classified into mono-rhamnolipids and di-rhamnolipids. At present, rhamnolipids are usually produced by various strains in the form of mixtures of diverse congeners, and the production strains, culture conditions, and carbon sources can affect the components of rhamnolipids [31,32]. Thus, RhlC is the key enzyme for di-rhamnolipid synthesis. Theoretically, *P. aeruginosa* strains merely synthesizing mono-rhamnolipids are achievable with a natural deficiency of *rhlC* or by knocking out *rhlC*. As shown in Table 2, several *Pseudomonas* strains had been reported to produce mono-rhamnolipids with the predominant congener as Rha–C_10_–C_10_. This underlines the truth that *P. aeruginosa* typically synthesizes various rhamnolipids containing fatty acids with a predominant C_10_ chain length. *P. aeruginosa* YM4 was an efficient rhamnolipids producer, with di-rhamnolipids (Rha–Rha–C_10_–C_10_) as a predominant component. However, we found that the expression of *irrE* of *D. radiodurans* in *P. aeruginosa* YM4 could make a difference in both the number of L-rhamnose residues and the fatty acids chain lengths of rhamnolipids, and mono-rhamnolipids congener Rha–C_10_–C_12_ was almost the sole component, accounting for 94.1% of total products. While we did not expect to synthesize mono-rhamnolipids, the global transcriptional regulator *irrE* we tried had exactly the same effect. It has been shown that the heterogenous global regulator *irrE* can affect the expression of thousands of genes in its hosts [20,33,34]. Therefore, the genome-wide transcriptional analysis of *irrE*-expressing strain XY01 at the stage of rhamnolipids accumulation should be carried out in future work.

## 4. Conclusions

Expression of *irrE* of *D. radiodurans* in *P. aeruginosa* YM4 increased its rhamnolipid production, as well as cell growth and cell tolerance to stress. Unexpectedly, mono-rhamnolipids congener Rha–C_10_–C_12_, accounting for about 94%, could be efficiently produced in the *irrE*-expression strain; the maximum titer reached 26 g/L using soybean oil and nitrate. This is the first report on altering the abundance and composition of rhamnolipids by the heterogenous expression of global regulator *irrE* in *P. aeruginosa*. This provides a new perspective for constructing efficient rhamnolipid-producing bacteria using synthetic biology techniques.

## 5. Materials and Methods

### 5.1. Strain, Medium, and Culture Conditions

Strain *Pseudomonas aeruginosa* YM4 (CCTCC M2017494) [24] was an efficient rhamnolipids producer and used as the starting strain for molecular manipulation. Table 3 provides a list of the strains and plasmids used in this work. All strains, except for those used in rhamnolipid production, were cultivated in LB medium containing peptone (10 g/L), yeast extract (5 g/L), and NaCl (10 g/L). Carbenicillin of 350 mg/L in LB medium was used for recombinant *P. aeruginosa* strains and ampicillin of 100 mg/L for *E. coli* strains.

The rhamnolipid fermentation medium consisted of soybean oil (50 g/L), NaNO_3_ (8 g/L), Na_2_HPO_4_ (7.888 g/L), KH_2_PO_4_ (1.972 g/L), CaCl_2_ (0.1 g/L), MgSO_4_ (0.2 g/L), NaCl (1 g/L), KCl (1 g/L), yeast powder (1 g/L), and a trace elements solution (2 mL/L) [24]. Strains were cultivated in 250 mL baffled flasks filled with 50 mL of medium and incubated at 37 °C with 200 rpm orbital shaking.

### 5.2. Construction of Reporter Plasmid

The broad host-range vector pHERD20T (5087 bp) was used as the backbone plasmid. However, the arabinose-inducible promoter P_BAD_ (located at 2303–3472 bp) present in the plasmid was replaced with the promoter of acyltransferase *rhlA* (P_rhlA_) in *P. aeruginosa* PAO1 (located at 3,893,009–3,893,431 bp of NC_002516.2, GenBank). To confirm the working effect of promoter P_rhlA_, we constructed expression cassette P_rhlA_-*gfp* in pHERD20T and the green fluorescent protein (GFP) was used as a reporter. The primers used in this study are listed in Table 4. Overlap PCR was employed to construct the P_rhlA_-*gfp* cassette. Firstly, the P_rhlA_ gene fragment was amplified from the genome of *P. aeruginosa* PAO1 using the primer pairs P_rhlA_-F/P_rhlA_-R·*gfp*, and the *gfp* gene was amplified from the genome of *B. subtilis* 168 (P_veg_-*gfp*) using the primer pairs *gfp*-F/*gfp*-R. Subsequently, fragments P_rhlA_ and *gfp* were fused together using the primers P_rhlA_-F/*gfp*-R to generate the P_rhlA_-*gfp* cassette. The resulting product was purified and sequenced. Next, the P_rhlA_-*gfp* cassette was joined to the linearized vector pHERD20T using TGGCGATAGCCCGGG and AAGCTTAGCTTGGCA as homology arms in a one-step cloning process. The arabinose operon region and the multi-cloning sites (located at 2303–3472 bp) were removed from the vector, resulting in the reporter plasmid pHERD20T-P_rhlA_-*gfp*. Sequencing was performed in the extracted reporter plasmid. The reporter plasmid was first transformed into *E. coli* TOP10 for amplification and then into strain YM4 for expression using chemical transformation methods as described in the literature [22].

### 5.3. Promoter Strength Measurements

The strength of the promoter was measured by the whole-cell fluorescence of recombinant strain YM4 (P_rhlA_-*gfp*) cultivated in LB medium and rhamnolipid fermentation medium using a fluorescence spectrophotometer (Hitachi Fluorescence Spectrophotometer F-7000, Hitachi, Naka, Japan). After culturing for 12 h, 1 mL of fermentation broth was centrifuged at 12,000 rpm for 10 min. The cells were collected and washed three times. The washed cells were resuspended in deionized water to the same optical density (OD_600_) 0.07. The excitation and emission wavelengths were 485 and 525 nanometers, respectively.

### 5.4. Construction of P_rhlA_-irrE Recombinant Plasmid

The GenBank accession numbers of *irrE*01, *irrE*02, and *irrE*03 are WP_010886813.1 (*D. radiodurans*), WP_014686212.1 (*D. gobiensis I-O*), and 3DTI_A (*D. deserti*), respectively; and all the three *irrE* genes were synthesized by GENEWIZ and sub-cloned into the pUC18 vector. The method described in 5.2 was also applicable to construct a P_rhlA_-*irrE* cassette of different origin and insert into the pHERD20T vector. The three recombinant expression plasmids pHERD20T-P_rhlA_-*irrE* were transformed into strain *P. aeruginosa* YM4, and finally the strain YM4 derivatives were named as strain XY01 (harboring *irrE*01), XY02 (harboring *irrE*02), and XY03 (harboring *irrE*03), respectively.

### 5.5. Extraction of Rhamnolipids and Determination of Strain Growth Rate

For seed culture, the strain YM4 and its derivants were inoculated into LB medium. After overnight incubation, 1 mL of the inoculum was transferred to each flask containing rhamnolipid fermentation medium. Strains were cultivated in 250 mL baffled flasks filled with 50 mL of medium and incubated at 37 °C with 200 rpm orbital shaking. The fermentation was carried out for 60 h. During fermentation, 500 μL culture fluid was taken for analysis of cell and rhamnolipid concentrations. The culture broth was mixed vigorously with *n*-hexane 1:1 (*v*/*v*) and centrifuged (12,000 rpm, 4 °C, 10 min) for separation of cells, and aqueous and *n*-hexane phases. The cell pellets were washed in deionized water (12,000 rpm, 4 °C, 10 min) and resuspended in deionized water for detection of the cell content at 600 nm. The aqueous phase was used for detecting the rhamnolipid content.

After fermentation, cell-free supernatants were collected for rhamnolipid extraction. The rhamnolipid products were precipitated and extracted using the method described in the literature [24].

The specific cell growth rate (μ, h^−1^) and specific rhamnolipid production rate (q_p_, h^−1^) of strain XY01 and strain YM4 were calculated using Equations (1) and (2).

(1) $$\mu =\frac{1}{x}\frac{dx}{dt}=\frac{1}{x}\underset{∆t\to 0}{\lim}\frac{∆x}{∆t}$$

(2) $$q_{p}=\frac{1}{x}\frac{dp}{dt}=\frac{1}{x}\underset{∆t\to 0}{\lim}\frac{∆p}{∆t}$$

### 5.6. Effect of IrrE on the Stress Tolerance of P. aeruginosa Cells

To explore whether the global regulator *irrE* of *D. radiodurans* could enhance the tolerance of *P. aeruginosa* cells to stress environments, spot tests were conducted. The seed cultures of strain XY01 and YM4 in LB medium were diluted to 3.0 at OD_600_, and 5 µL aliquots of a tenfold dilution series were spotted onto LB agar plates supplemented with 10 g/L or 20 g/L rhamnolipid and the pH adjusted to 7.0, 7.5, and 8.0 with 2M NaOH solution. The plates were incubated at 37 °C for 12 h. The experiments were repeated twice.

### 5.7. Quantitative Analysis of Rhamnolipids

Rhamnolipids were detected directly by HPLC using an evaporative light scattering detector (ELSD). As described in the literature [24], rhamnolipid samples were prepared; the equipment used was a 1260 Infinity HPLC-ELSD (Thermo Scientific Ultimate 3000 HPLC, Palo Alto, CA, USA)−(Alltech ELSD2000ES, Chicago, IL, USA) equipped with a C18 column (4.6 × 150 mm, 5 µm; Sepax Technologies, Inc., Suzhou, China). The ELSD was set up with a drift temperature of 103 °C and a nebulizer flow rate of 2.8 L/min. A linear gradient from 30% to 100% of acetonitrile in 40 min was used with mobile phase A (acetonitrile) and mobile phase B (water with 0.05% formic acid) at the flow rate of 1 mL/min. Rhamnolipids standard (R90, Lot number: A79125126058) was obtained from AGAE Technologies (Corvallis, OR, USA); and standard curves comprising 0.1 to 1 mg/mL of rhamnolipids were used for quantification. The area normalization method was used to determine relative content of rhamnolipids congeners.

### 5.8. Characterization of Rhamnolipids

#### 5.8.1. HPLC-MS Analysis of Rhamnolipids

The sample preparation, C18 column, and the parameters of the mobile phase were the same as those in 5.7. The mass spectrometry analysis was performed using an Agilent 6530 Q-TOF (Agilent Technologies 6530 Accurate-Mass Q-TOF LC/MS, Palo Alto, CA, USA) instrument with electrospray ionization (ESI) in positive ion mode. The capillary voltage was set to 4 kV. The gas temperature was 350 °C. The drying gas flow rate was 5 L/min. The Oct 1 RF Vpp was set to 750 V. The mass spectrum scanning range (*m*/*z*) was 50–2000.

#### 5.8.2. Fourier-Transform Infrared (FTIR) Analysis of Rhamnolipids

The spectral analysis of the purified XY01 rhamnolipids was performed using FTIR spectroscopy, determining the significant chemical bonds present in the fingerprint area. An infrared spectrophotometer (Thermo Scientific Nicolet iS5, Waltham, MA, USA) operating in the attenuated total reflection (ATR) mode was used to collect the spectra with a resolution of 4 cm^−1^ from 400 to 4000 wavenumbers (cm^−1^).

#### 5.8.3. Determination of the Critical Micelle Concentration (CMC)

The CMC values were determined by measuring the changes of surface tension with different concentrations of rhamnolipids. The surface tension of rhamnolipid solutions with varying concentrations (0–200 mg/L) was measured at room temperature using an automated tensiometer (BZY-3B, Shanghai Automation Instrumentation Sales Center, Shanghai, China).

#### 5.8.4. Determination of the Emulsification Index (EI_24_)

The emulsifying activity of rhamnolipids produced by strain XY01 and strain YM4 were comparatively evaluated using the emulsification index (EI_24_). Diesel oil was used as the organic phase in this test. Briefly, 2 mL of diesel oil and 2 mL of rhamnolipid sample were vortexed at high speed (~2800 rpm) for 2 min in 5 mL stoppered glass tubes. These mixtures were then left to stand at room temperature for 24 h. The EI_24_ was calculated by dividing the measured height of the emulsion layer (mm) by the total height of the mixture (mm) and multiplying by 100.

## Figures and Tables

**Table 1:** Structural composition of rhamnolipids produced from strains YM4, XY01, XY02, and XY03.

Peak	Retention Time (min)	Structure	Molecular Weight	Relative Abundance (%)				
				Standard *	YM4	XY01	XY02	XY03
1	4.87	NA	NA	2.99	-	-	-	-
2	5.53	NA	NA	3.11	-	-	-	-
3	10.56	Rha_2_–C_10_–C_10_	650	46.52	58.72	-	56.14	57.89
4	13.28	Rha–C_10_–C_10_	504	41.88	38.86	-	41.22	40.29
5	14.87	NA	NA	2.22	1.33	-	1.11	0.78
6	16.28	Rha–C_10_–C_12_	532	2.11	0.64	94.14	0.52	0.58
7	19.04	NA	NA	1.17	0.45	1.19	1.01	0.37
8	11.95	NA	NA	-	-	2.89	-	-
9	22.33	NA	NA	-	-	0.78	-	-

* Rhamnolipids standard (R90, Lot number: A79125126058) was obtained from AGAE Technologies (Corvallis, OR, USA). NA: not available.

**Table 2:** Mono-rhamnolipid-producing strains and characteristics.

Strians	Genetic Characteristics	Carbon Source	Titer (g/L)	Mono-Rhamnolipid Congeners (Titer Ratio,%)	Reference
*P. putida* KT2440	Expressing the *rhlAB* operon in *P. putida* KT2440	Glucose	2.4	Rha–C_8_–C_10_; 2% Rha–C_10_–C_10_; 63% Rha–C_10_–C_12:1_; 16% Rha–C_10_–C_12_; 19%	[35]
*P. aeruginosa* SG ΔrhlC	Knocking out *rhlC* in *P. aeruginosa* SG	Glycerol	14.22	Rha–C_10_; 3.4% Rha–C_8_–C_10_; 10.9% Rha–C_10_–C_10_; 25.9% Rha–C_10_–C_12:1_; 43.5% Rha–C_10_–C_12_; 16.4%	[36]
*P. aeruginosa* ATCC 15442	Natural deficiency of *rhlC*	Glucose	NA *	Rha–C_8_–C_10_; NA Rha–C_10_–C_10_; NA Rha–C_10_–C_12:1_; NA Rha–C_10_–C_12_; NA	[37]
*P. aeruginosa* CR1	Natural deficiency of *rhlC*	Glycerol	21.77	Rha–C_8_–C_10_; NA Rha–C_10_–C_10_; NA Rha–C_12:2_–C_10:1_; NA	[38]
*P. aeruginosa* XY01	Expressing *irrE* of *D. radiodurans* in *P. aeruginosa* YM4	Soybean oil	26	Rha–C_10_–C_12_; 94.1%	This work

* NA: not available.

**Table 3:** Strains and plasmids used in this study.

Strains and Plasmids	Characteristics	Sources
**Strains**		
*P. aeruginosa* YM4	Rhamnolipid-producing strain (original strain)	This lab
*P. aeruginosa* PAO1	Source of promoter P_rhlA_	This lab
*B. subtilis* 168 (P_veg_-*gfp*)	Source of *gfp*	This lab
*E. coli* TOP10	Plasmid amplification in vivo	This lab
*P. aeruginosa* XY01	Strain YM4 derivative; harboring pHERD20T-P_rhlA_-*irrE*01	This work
*P. aeruginosa* XY02	Strain YM4 derivative; harboring pHERD20T-P_rhlA_-*irrE*02	This work
*P. aeruginosa* XY03	Strain YM4 derivative; harboring pHERD20T-P_rhlA_-*irrE*03	This work
**Plasmids**		
pHERD20T	Broad host-range vector; pUCP20T P_lac_ replaced with 1.3 kb AflⅡ-EcoRⅠ fragment of araC-P_BAD_ cassette (5087 bp)	Provided by Dr. Xu Anming @ Nanjing Tech University
pHERD20T-P_BAD_-*gfp*	pHERD20T P_BAD_ fused with *gfp* gene	This work
pHERD20T-P_rhlA_-*gfp*	Reporter plasmid; pHERD20T P_BAD_ replaced with P_rhlA_ and fused with *gfp* gene	This work
pHERD20T-P_rhlA_-*irrE*01	pHERD20T P_BAD_ replaced with P_rhlA_ and fused with *irrE* gene from *D. radiodurans*	This work
pHERD20T-P_rhlA_-*irrE*02	pHERD20T P_BAD_ replaced with P_rhlA_ and fused with *irrE* gene from *D. gobiensis* I-O	This work
pHERD20T-P_rhlA_-*irrE*03	pHERD20T P_BAD_ replaced with P_rhlA_ and fused with *irrE* gene from *D. deserti*	This work

**Table 4:** Primers used in this study.

Premier ID	Sequence (5′→3′)
pHERD20T-F	AAGCTTAGCTTGGCACTGGCCGTCG
pHERD20T-R	CCCGGGCTATCGCCACCGTCG
P_rhlA_-F	TGGCGATAGCCCGGGCGCCAGAGCGTTTCGACA
P_rhlA_-R·*gfp*	CTCCTTTTGACATTTCACACCTCCCAAAAATTTTCGAACAG
*gfp*-F	TGGGAGGTGTGAAATGTCAAAAGGAGAAGAACTTTTTACAGGTG
*gfp*-R	CGGCCAGTGCCAAGCTAAGCTTTTATTTATAAAGTTCGTCCATACCGTG
P_rhlA_-R01	TCGGCATTTCACACCTCCCAAAAATTTTCGAACAG
*irrE*01-F	TTTGGGAGGTGTGAAATGCCGAGCGCGAATGTTTCA
*irrE*01-R	TGCCAAGCTAAGCTTTCACTGTGCCGCATCTTGCGGTTCAT
P_rhlA_-R02	TGCCAGTTCTCTCATTTCACACCTCCCAAAAATTTTCGAACAG
*irrE*02-F	AGGTGTGAAATGAGAGAACTGGCAGCGGCGTAT
*irrE*02-R	TGCCAAGCTAAGCTTTCATGTGCCTCCTCTGCCAT
P_rhlA_-R03	CGGATCTGTCATTTCACACCTCCCAAAAATTTTCGAACAG
*irrE*03-F	TTTGGGAGGTGTGAAATGACAGATCCGGCGCC
*irrE*03-R	TGCCAAGCTAAGCTTTCAGCTCTGATCTCCCGGT

## Data Availability

Data are contained within the article and Supplementary Materials.

## Supplementary Materials

The following supporting information can be downloaded at: https://www.mdpi.com/article/10.3390/molecules29091992/s1, Data S1: Supplementary data.
