# Supplementary material for: A Rare Mono-Rhamnolipid Congener Efficiently Produced by Recombinant Pseudomonas aeruginosa YM4 via the Expression of Global Transcriptional Regulator irrE
Source: Molecules. 2024 Apr 26;29(9):1992. doi: 10.3390/molecules29091992 (PMC11085080; doi:10.3390/molecules29091992)
Supplement: Supplementary file 1 [file molecules-29-01992-s001.zip › molecules-2936311-supplementary.pdf]

Supplementary data

Sequence of the original promoter of rhlA (P_{rhlA}) and its RBS located at 3893009-3893431 bp of NC_002516.2, GenBank.

CGCCAGAGCGTTTCGACACCGGAAACCGGGCCTGGCGCCCGGTTTTTCATGCCTTTT
CCGCCAACCCCTCGCTGTTCCCCGCCGGCCGCTCTGGCACGCCTTATCGCGGGCGGGC
AGGGGCTTATGCGCAGGCGGCCGCCCGTCCTGTGAAATCTGGCAGTTACCGTTAGCTT
TCGAATTGGCTAAAAAGTGTTCATCGGCTACGCGTGAACACGGACGCCAATCGTTTGC
GCAGGCCGATCTGCAAGACCCACACAAGCCCCTCGCCTGAAGGGGTACGCATCCGCC
GTGGCTGGTCCGCGCGGATGGCCGCTGAGTTACTTGTCTGCCGTTCGAACAATAAGAA
CGAACTCTACGTAATGCCGGGATACCCGTGGCAGCGATAGCTGTTTGCCTGTTCGAAAA
TTTTTGGGAGGTGTGAA

ORF of *irrE*01:

MPSANVSPPCPSGVRGGGMGPKAKAEASKPHPQIPVKLPFVTAPDALAAAKARMRDLAA
AYVAALPGRDTHSLMAGVPGVDLKFMPLGWRDGAFDPEHNVILINSAARPERQRFTLAH
EIGHAILLGDDDLLSDIHDAYEGERLEQVIETLCNVAAAAILMPEPVIAEMLERFGPTGRAL
AELAKRAEVSASSALYALTEQTPVPVIYAVCAPGKPPREQAASDEDAGPSTEKVLTVRASS
STRGVKYTLASGTPVPADHPAALALATGMEVREESYVPFRSGRKMKAEVDAYPSRGIVAV
SFEFDPARLGRKDSEQADRDEPQDAAQ

ORF of *irrE*02:

MRELAAAYAARVPSLDAHGLMDGLDGVQLRFMPMGQRDGAYDPEHHVILINSQVRPER
QRFTLAHEISHALLLGDDDLLSDLHDSFEGERLEQVIETLCNVGAAALLMPDALIAELLER
FGATGRALAELSRRADVSASTALYALAERTPGAVLYAVCTRSRLETETDDEDGGAASGTAL
TVRVSGGSAGMKYTLRPGTPIPADHPVQAAFESNLPLTGPSYVPFRSGRKMPAEVDAFPVR
GRVMVSFDLNGRGGT

ORF of *irrE*03:

MTDPAPPPTALAAAKARMRELAASYGAGLPGRDTHSLMHGLDGITLTFMPMGQRDGAY
DPEHHVILINSQVRPERQRFTLAHEISHALLLGDDDLLSDLHDEYEGDRLEQVIETLCNVG
AAALLMPAELIDDLLTRFGPTGRALAELARRADVSATSALYALAERTAPPVIYAVCALSRQ
EDEGEGGGAKELTVRASSASAGVKYSLSAGTPVPDDHPAALALDTRLPLAQDSYVPFRSG
RRMPAYVDAFPERQRVLVSFALPAGRSEPDADKPEAPGDQS
